# A Comparison Study of Ag Composites Prepared by Spark Plasma Sintering and Hot Pressing with Silver-Coated CNTs as the Reinforcements

**DOI:** 10.3390/ma12121949

**Published:** 2019-06-17

**Authors:** Dongming Jia, Junbing Ma, Xueping Gan, Jingmei Tao, Ming Xie, Jianhong Yi, Yichun Liu

**Affiliations:** 1 School of Materials Science and Engineering, Kunming University of Science and Technology, Kunming 650093, China; Jiananom@sina.com (D.J.); Compositekmust@163.com (J.M.); Taocomposite@sina.com (J.T.); Yikmust@126.com (J.Y.); 2 State Key Laboratory of Powder Metallurgy, Central South University, Changsha 410083, China; 13629490793@163.com; 3 Kunming Institute of Precious Metals, Kunming 650106, China; Xiekmust@126.com

**Keywords:** carbon nanotubes, silver matrix composites, electroless deposition, spark plasma sintering, hot pressing

## Abstract

In this study, carbon nanotube-reinforced silver composites (CNT/Ag) were prepared by the powder metallurgy process via spark plasma sintering (SPS) and hot pressing sintering (HP) with composite powders through an improved electroless plating method assisted by ultrasonic spray atomization. The dispersion of CNTs was effectively improved by ultrasonic spray atomization, and uniform silver layers were deposited on the surface of CNTs by electroless deposition. The property testing results showed significant improvements of the electrical conductivity, hardness, and tensile strength in the samples prepared by SPS, as compared to their HP sintered counterparts. When the volume fraction of CNTs reached 2.5%, the tensile strength reached a maximum value of 221 MPa, which was more than twice that of the pure silver samples. The structural analysis indicated different degrees of CNT agglomeration and matrix mean grain sizes in the composites prepared by SPS and HP, which are responsible for the differences in properties.

## 1. Introduction

Since carbon nanotubes (CNTs) were discovered, a significant amount of research has been conducted to exploit their properties [1,2]. Theoretical and experimental studies have shown that these materials possess great mechanical properties, excellent toughness, and superior physical properties [3,4,5,6]. There are many kinds of reinforcing phases of silver matrix composites, and the properties of the composites primarily depend on the properties, content, distribution, and dispersion of the reinforcing phase [7]. CNT silver matrix composites have many special properties that neither pure silver nor silver alloys have. CNTs have many advantages, including a stable size, high specific strength and modulus, easy machining, and good thermal conductivity, among others. They are widely used in many fields, such as field launch, medical treatment, power transmission, printing, and soldering [8,9]. However, the production of CNT-Ag composites is severely limited due to both the dispersion of CNTs in the silver matrix and the interfacial binding force between CNTs and silver.

To enhance the interfacial bonding, CNT-metal powders are usually prepared via physical evaporation, magnetron sputtering, and electroless plating. Of these methods, electroless plating is particularly intriguing because of its low cost, inherent selectivity, and simplicity [10,11,12]. In this study, silver was deposited on the surface of CNTs by electroless plating assisted by ultrasonic spray atomization (EPUSA) to improve the interfacial bonding between the CNTs and the silver matrix. The coated layers of silver can then serve as a medium layer for adhesion improvement and load transferring [13,14]. 

For this paper, the effects of EPUSA on the dispersion of CNTs and the properties of composites sintered by SPS and HP were studied. The improved electroless plating method that we adopted allows for the realization of the process on a micrometer scale. This achieves the preparation of a layer of uniform silver nanoparticles on the surface of the CNTs. To obtain a higher uniformity of CNT deposits on the Ag matrix, the produced CNTs coated with Ag nanoparticles (Ag@CNTs) were mixed with silver powders via solution ball milling (SBM) to obtain composite powders (Ag@CNTs /Ag). Then the Ag@CNTs/Ag composites were prepared by SPS and HP. In both cases, a cylindrical graphite die is filled with a powder bed. A uniaxial macroscopic compaction pressure is then applied via graphite punches to the constrained powder bed, which is simultaneously exposed to a high temperature. The high-temperature source is the primary difference between HP and SPS. In HP, a high temperature is obtained by resistance heating elements surrounding the die. In the case of SPS, a pulsed direct current is sent to the powder bed. As a novel sintering technique, the SPS technique can implement extremely fast cooling rates, and is characterized by a short holding time at a relatively lower sintering temperature, which might be appropriate for the fabrication of the desired composites in the present study. In this paper, the influence of the sintering mode on the distribution of CNTs is discussed. The effect of the distribution state of CNTs on the enhancement of carbon nanotube-silver composites was investigated and discussed from the aspects of both electrical and mechanical properties. 

## 2. Experimental Section

**Figure 1:**
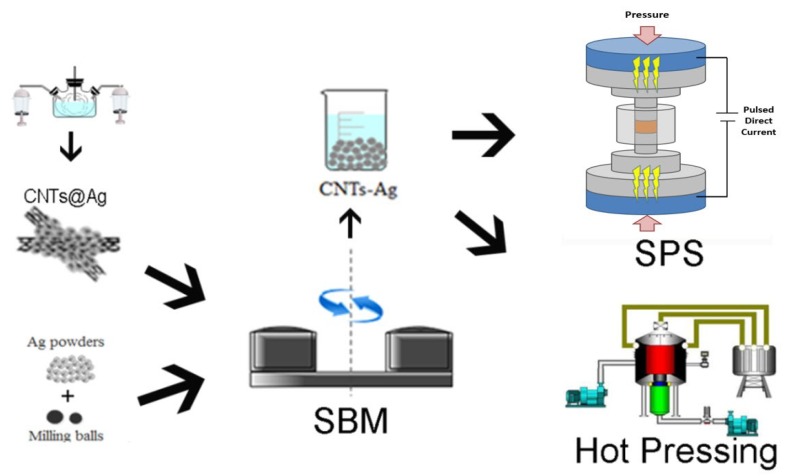
Experimental flow chart for synthesis of CNTs-Ag composites. (SBM: solution ball milling).

Figure 1 is an experimental flow chart of the synthesis progress of CNT-Ag composites. First, a layer of silver nanoparticles was deposited onto the pretreated CNT surfaces by ultrasonic spray atomization. The composite powders of the modified CNTs and silver powder were then obtained by ball milling. Finally, the composite materials were prepared by sintering the composite powders via HP and SPS.

### 2.1. Synthesis of Ag@CNTs

The silver deposited on the CNT surfaces was obtained by electroless plating assisted by ultrasonic spray atomization (EPUSA). Commercial Ag powder (purity ≥ 99%) and multi-walled CNTs (inner diameters of 5–12 nm, outer diameters of 20–50 nm, lengths of 10–20 µm, purity ≥ 95 wt.%) were acquired from Time Nano Co., Ltd. of China (Chengdu, China). Pretreatments including oxidation, sensitization, and activation were performed before the electroless plating, as described by Zhao et al. [15]. After carrying out the pretreatment processes, the CNTs were dispersed in a 200 mL hydrazine hydrate solution by mechanical and ultrasound agitation to get the suspension Solution B. Solutions A (0.006 M AgNO_3_ and 0.115 M NH_3_·H_2_O) and B were prepared at a 1:1 (volume) ratio. In the EPUSA process, Solutions A and B were respectively atomized to droplets with ultrasonic atomizers, and the droplets were then delivered to a three-mouth flask for the upcoming solution mixture and electroless plating for 5 min at 293 K. The gained precipitates were dried at 323 K in a vacuum drying oven to obtain the Ag@CNTs powders. Further details on the preparation of the Ag@CNTs powders can be found in our previous reports [13,14].

### 2.2. Spark Plasma Sintering and Hot Pressing of Composites

Two kinds of composite powders of CNTs/Ag and Ag@CNTs /Ag were produced by SBM. In the process, different contents Ag@CNTs were mixed with Ag powders, put into a planetary ball mill using pure ethanol as the liquid medium, and then milled for 2 h under an air atmosphere condition. The rotation speed was 300 rpm in a one-way manner, and the ball to powder ratio was 10:1. After filtering and drying, a series of flake alloy powders with different volume fractions of CNTs was obtained. The mixed powders were then dried at 333 K for 12 h in a vacuum oven. Afterwards, the mixed powders were put into a cylindrical graphite die with an inner diameter of 20 mm, and were sintered by SPS (SPS-330, Fuji Radio Corp., Chiryu City, Japan) and HP ((HIGH-MULTI-5000, Fuji Radio Corp., Chiryu City, Japan). SPS sintering was conducted at 973 K with a pressure of 50 MPa in a vacuum condition for 5 min, and the heating rate was 100 K/min. The sintering temperature by HP was the same as that by SPS, and had a heating rate of 20 K /min and was maintained at 973 K for 30 min under the same conditions as SPS.

### 2.3. Characterization

The samples were characterized with a metalloscope (Kingsalee 9-21, Carl Zeiss Corp., Jena, Germany), scanning electron microscopy (SEM, FEI NovaNano-450, FEI Corp., Hillsboro, OR, USA), transmission electron microscopy (TEM, Tecnai G2 TF30 S-Twin, FEI Corp., Hillsboro, OR, USA), and Raman spectroscopy (LabRAM HR Evolution, HORIBA JOBIN YVON Corp., Kyoto, Japan). Electrical conductivities were measured with an eddy current conductivity meter (Sigma2008B, Xiamen Tianyan Instrument Co., Ltd., Xiamen, China), and the values of Vickers hardness were measured using a micro hardness tester (HVST-1000Z, Shanghai Research Runguang Technology Co., Ltd., Shanghai, China) with a load of 0.98 N for 15 s, and the measurement was carried out 30 times for each sample. 

## 3. Results and Discussion

### 3.1. Microstructure of Ag@CNTs

**Figure 2:**
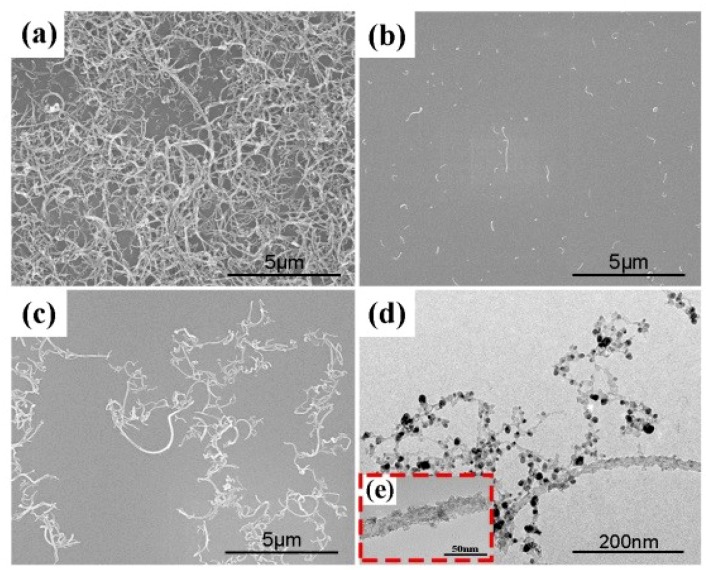
Effects of EPUSA on the dispersion of CNTs, (**a**) the CNTs before EPUSA; (**b**) CNTs of 2 min with EPUSA; (**c**) CNTs of 5 min with EPUSA; (**d**) TEM image of Ag@CNTs composites prepared by EPUSA and (**e**) TEM image of Ag@CNTs.

To demonstrate the dispersion improvement of CNTs via ultrasonic spray or EPUSA, CNTs were put into deionized water and then ultrasonic spray was applied. Silicon substrates were used to collect the droplets for 2 min and 5 min, respectively and then dried for SEM observation. The dispersion of original CNTs is presented in Figure 2a and the dispersion of ultrasonic-spayed CNTs was effectively improved as shown in Figure 2b,c, as a result of the ultrasonic agitation and the limited volume of each droplet. The good dispersion will be maintained throughout the EPUSA reaction process. The excellent dispersion of CNTs is an important basis for the uniform nano silver coating deposited on their surfaces. Additionally, the uniform nano silver coating deposited on CNTs can prevent the agglomeration of CNTs in the reaction process. To obtain uniform and continuous silver coating, a reaction time of 5 minutes was used in this study. From the microstructures displayed in Figure 2d,e, it can be seen that the silver metal was deposited on the CNT surfaces in a coated type morphology. The Ag@CNTs composite powders are relatively decentralized, as can be seen in Figure 2d, which is beneficial for solving the problem of the distribution of CNTs in the Ag matrix via the SBM process. 

### 3.2. Microstructure of the Powders and Composites

**Figure 3:**
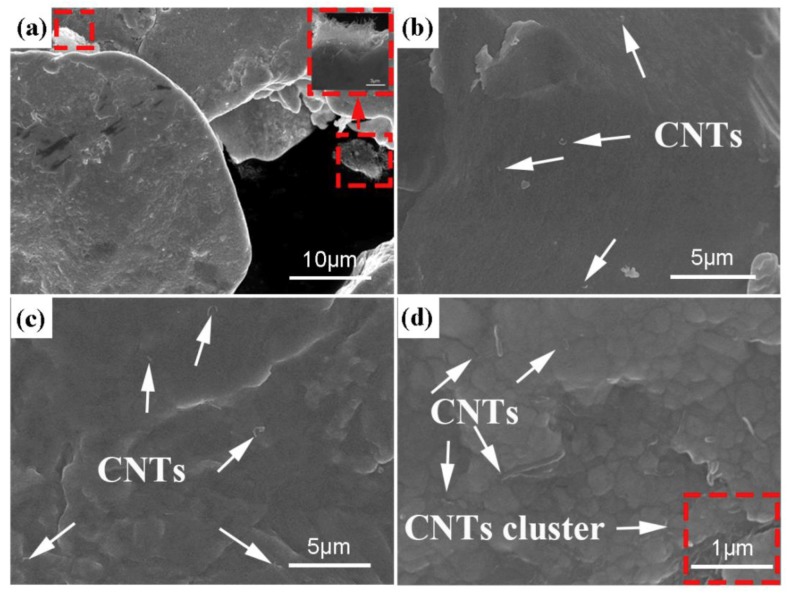
SEM images of (**a**) CNTs/Ag composites powders (2.5 vol.%) and CNTs@Ag/Ag powders mixed by solution ball milling process: (**b**) 1 vol.%, (**c**) 2.5 vol.% and (**d**) 5 vol.%.

Figure 3a reveals that some CNT agglomerations exist on the silver powders. The CNT agglomerations have not been dispersed under the impact of the milling balls during ball milling progress. Figure 3b–d show the surface morphologies after SBM of the Ag@CNTs /Ag composite powders with CNT contents of 1 vol.%, 2.5 vol.%, and 5 vol.%, respectively. It can be clearly seen from Figure 3b,c that the CNTs coated with Ag are uniformly dispersed in the silver matrix without agglomeration, and that the Ag@CNTs exist in an embedded form. Furthermore, by increasing the content of Ag@CNTs, the CNT volume content was increased; as can be seen in Figure 3d, when CNTs reached up to 5 vol.%, a certain amount of agglomerates appeared in the silver matrix. In our previous work, it was found that CNT aggregates still exist in the form of aggregates after sintering, and these aggregates indeed reduce the properties of matrix composites. Therefore, Ag@CNTs obtained by EPUSA was used in the later research to explore the influence of the sintering mode on the composite properties of materials. 

**Figure 4:**
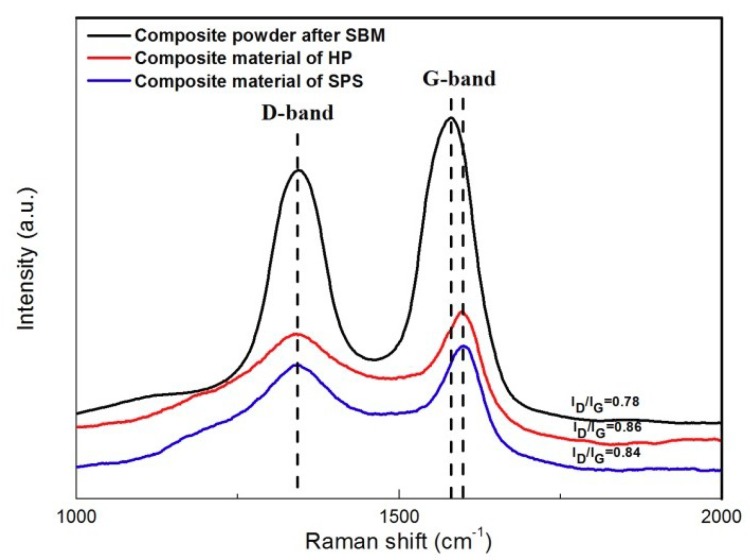
Raman spectra of: composite powder and composite material of HP and SPS.

The structural changes of CNTs after sintering were detected by Raman spectra, which are presented in Figure 4. Two features in the first-order Raman spectra are a G-band at 1570–1580 cm^−1^, indicating two-dimensional graphitic ordering of nested graphene layers of McCants, and a D-band at 1340–1350 cm^−1^, which is highly responsive to the non-planar atomic distortions, amorphous carbon, CNT curvature, and other carbon impurities. It can be seen from the results that the intensity ratio of the D to G modes basically did not change after sintering, indicating that the structural integrity of the CNTs was well preserved. Slightly different from the case of composite powders after SBM, the G-band of CNTS in the composite material slightly shifted to a higher wavenumber, and such a peak shift has been attributed to the structural change of CNTs during processing [16,17], the bonding condition, and even the infiltration of metal atoms in CNTs [18,19]. 

**Figure 5:**
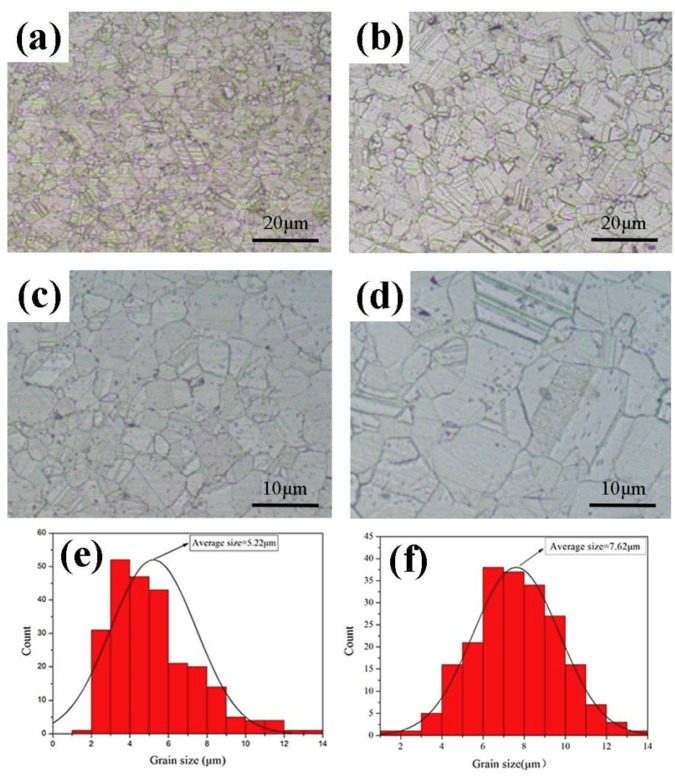
Metallographic observations and statistical charts of the composites: (**a**,**c**,**e**) for SPS, (**b**,**d**,**f**) for HP.

Figure 5 presents the metallographic images of the composite material with a CNT content of 2.5 vol.% respectively prepared by HP and SPS. Silver grain sizes were estimated by averaging the length and width of at least 200 grains in the metallographic observation. The particle size distributions are shown in Figure 5e,f. The average particle size increased from 5.22 μm to 7.62 μm, exhibiting an increase of about 45.9%. Moreover, the particle size range of the composite materials obtained by SPS is mostly distributed in the small particle size range, and the structure is even. It is generally believed that grain refinement will contribute to strengthening and toughening of the materials [20].

**Figure 6:**
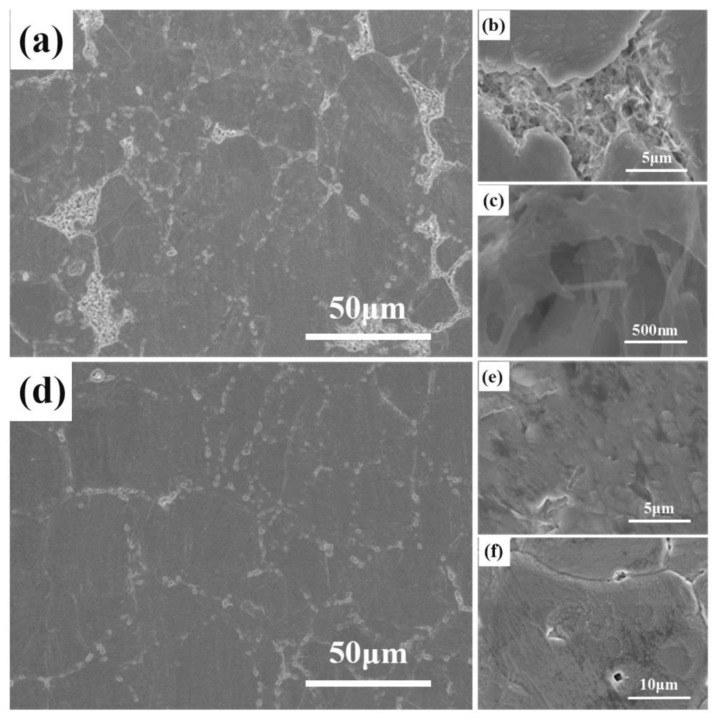
SEM images of CNTs @ Ag/Ag composites microstructure, (**a**–**c**) by HP, (**d**–**f**) by SPS.

Figure 6 displays the SEM images of the 2.5 vol.% Ag@CNTs/Ag materials respectively prepared by SPS and HP. As can be seen from Figure 6a–c, there is a large number of CNT aggregates in the sample prepared by HP. The presence of these aggregates obviously reduces the performance of the sample. Figure 6d shows that there are no obvious aggregates in the sample prepared with SPS, and the CNTs are relatively well dispersed at the grain boundary of the Ag matrix. The enlarged images in Figure 6e,f clearly show that the CNTs are dispersed separately in the matrix. 

**Figure 7:**
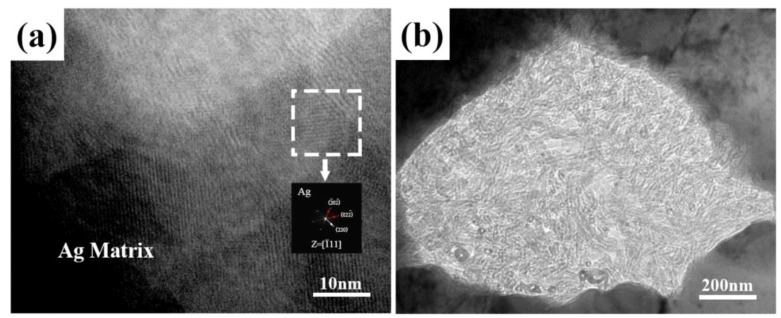
TEM images of CNTs @ Ag/Ag composites microstructure, (**a**) by SPS, (**b**) by HP.

Figure 7 presents the TEM images of the microstructures of the Ag@CNTs/Ag composites. It can be seen from Figure 7a that silver exists around the CNTs, including at the interface of the CNTs and the Ag matrix. This is because the silver deposited on the surface of the CNTs has diffused into the silver matrix during the sintering process. Some of the silver has spread at the interface of the CNTs and the silver matrix, which improved the interface bonding between them. This is one of the important reasons for improving the mechanical properties of the samples sintered by SPS. However, it can be seen in Figure 7b that there is a large amount of agglomerated CNTs at the grain boundary, which is also consistent with the result of SEM in the front. The reason for this is that the grains grew with the increase of the heat preservation time during HP, resulting in grain boundary migration and CNT agglomeration. This is also a key factor that led to the poor mechanical properties of the sample sintered by HP. In our previous work, we have found that there is an interface zone between the Ag matrix and the CNT reinforcement that has a thickness of about 2–4 nm. Moreover, the amorphous layer of CNTs exhibits good wettability with the Ag matrix. The Ag matrix and Ag nanoparticles are in parallel, and coherent interfaces are formed through the (111) plane. 

### 3.3. Mechanical Properties of the Composites

**Figure 8:**
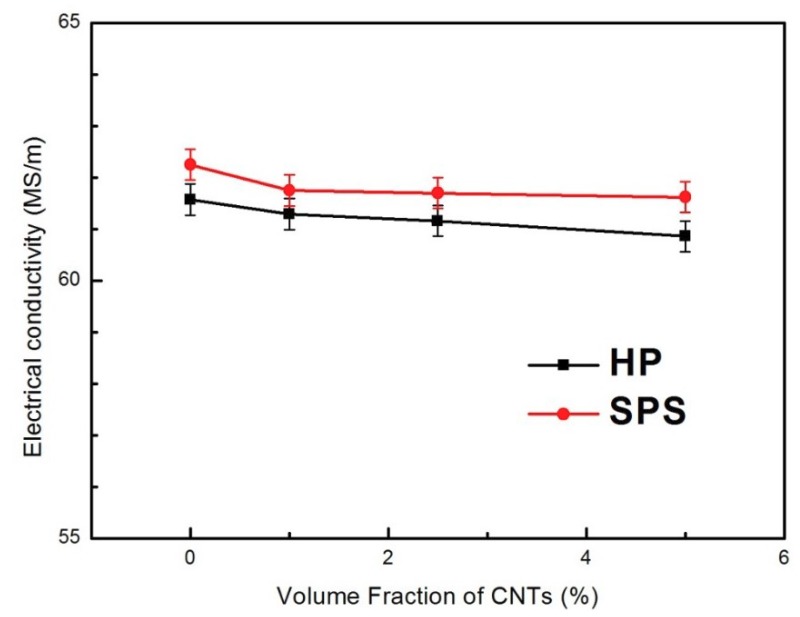
Electrical conductivity of the CNTs@ Ag/Ag composites prepared via SPS and HP.

Figure 8 shows the electrical conductivity of the composites with different CNT content. Compared with the hot-pressing sintering process (HP), the conductivity of the CNT-Ag composite prepared by spark plasma sintering (SPS) ranges from 62.25 to 61.62 MS/m, with no significant decrease (in Figure 8). As a reference, the standard conductivity of sterling silver is 63.01 MS/m. The difference in the electrical conductivity of the composites at the same CNT volume percentage using different sintering methods can be explained as follows. First, when the SPS sintering method is used, the Ag@CNTs are evenly distributed on the grain boundary, and form a network that contributes to the phase continuity of CNTs and provides an interconnected electrical path for the reduction of resistivity [21]. Second, when the HP sintering method is used, the formation of a large number of Ag-CNT aggregates not only hinders the densification of samples, but also becomes the source of defects, which leads to the reduction of the conductivity of the composites. Moreover, the silver nanoparticles deposited on the surface of Ag@CNTs play an important role in reducing the resistivity by filling and connecting the empty areas of the silver matrix. On the other hand, electron scattering at the strong interface that formed between the Ag-CNTs and the Ag substrate is also important to the electronic conductivity. The negative effect on electron transport is offset by the low boundary resistance combined with the strong interface [22]. 

Furthermore, with increasing CNTs, the conductivity of composites exhibits a gradually decreasing trend. This is because the electron scattering of CNTs at a high volume percentage will decrease the electrical conductivity. The high surface area of the CNTs results in a larger volume of the interface between the CNT-Ag composite, leading to greater scattering in the electron transfer process, and thus increasing the resistivity of the composites [9]. 

**Figure 9:**
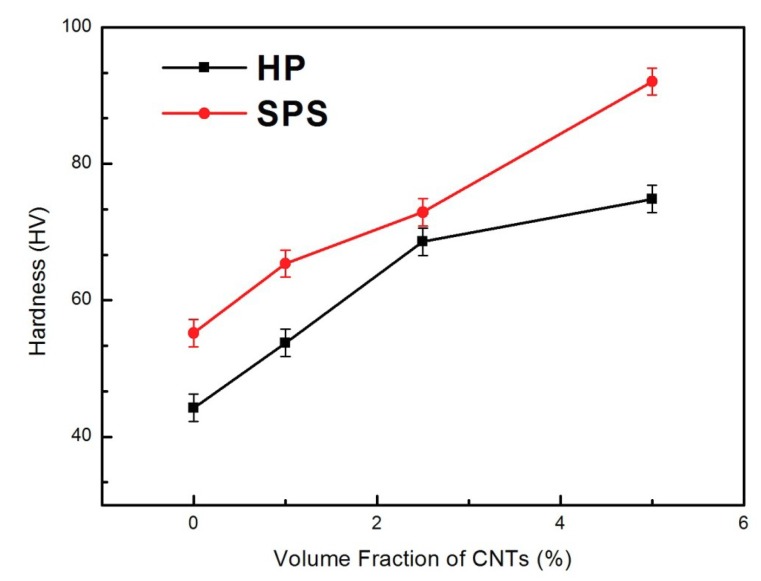
Hardness of the CNTs@Ag/Ag composites prepared via SPS and HP.

Figure 9 shows the hardness of the composites. A significant enhancement in hardness is observed when CNTs are incorporated into the Ag matrix. The hardness values increase almost linearly with the CNTs content. When the volume fraction of CNTs reached 5%, the hardness values of the two series of composites are 74.83 and 92.05, respectively, which is about 69.11% and 66.73% more than that of the pure silver sample. This is mainly because CNTs have a higher stiffness and strength than does the Ag matrix, and the uniform dispersion of CNTs in the collective can effectively improve the mechanical properties. However, at the same volume percentages of CNTs in the two series of composites, the significant difference in the hardness of the composites may be explained as follows. First, CNTs mostly exist as aggregates in composites prepared by the HP process. The agglomeration of CNTs results in the reduction of the compactness of the composites, which leads to the reduction of hardness. Second, the improved interfacial strength of Ag@CNTs and the embedding distribution can inhibit the matrix deformation. The uniform dispersion of Ag@CNTs within grain boundaries can also inhibit nucleation and motion of dislocation to a greater extent, leading to the improvement of mechanical properties [23]. The results demonstrated that the significant increase in hardness was derived from the uniform distribution of Ag@CNTs in the Ag matrix, and the interface strength of the CNT-Ag interface. Therefore, based on these results, when the uniformly distributed CNTs in the Ag matrix share the external loads, it can be expected that the mechanical properties of the Ag matrix enhanced by CNTs will be improved.

**Figure 10:**
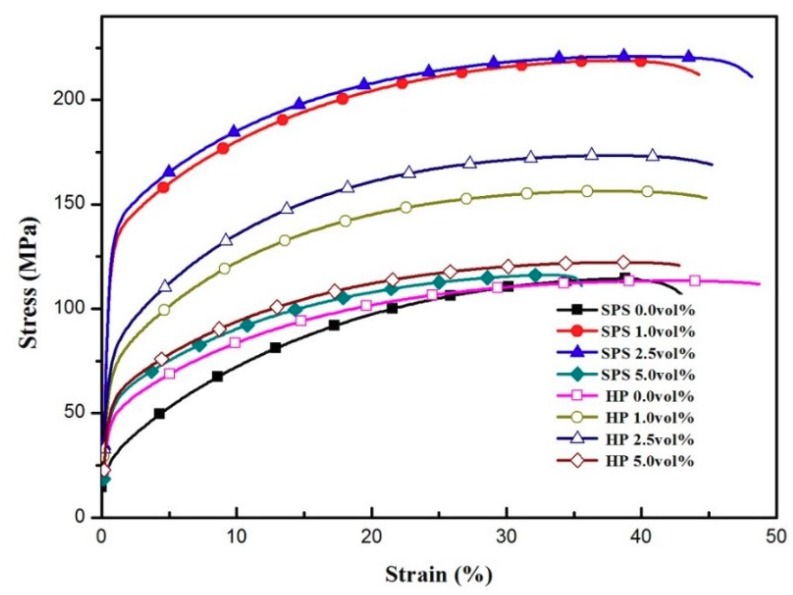
Stress-strain curves of the CNTs@Ag/Ag composites.

The engineering stress-strain curves of the CNTs@Ag/Ag composites are presented in Figure 10. The loading direction is parallel to the compaction direction of the samples. The tensile strength of the composite material with a volume fraction of 2.5% is 220 MPa, about twice that of pure silver sintered by SPS. However, in 5 vol.% Ag@CNTs/Ag composites, the tensile strength decreases to 150 MPa, which is only about 1.3 times that of pure silver. In the tensile strength test, when the volume fraction of CNTs increased to more than 2.5%, the accumulation of CNTs in the silver matrix reduced the tensile property and enhanced the crack expansion. The results also show that the elongation of the composites decreased when the volume fraction of CNTs increased to 5%. The elongation of pure silver is about 48%, while the content of 5 vol.% CNTs decreased this to 35%. When the contents of CNTs were the same, the tensile strength of the composites prepared by SPS was much higher than that of the composites prepared by HP. This can be explained by the following factors. First, the strengthening mechanism of CNT reinforcement is believed to be related to the unique structural characteristics and the excellent mechanical properties of CNTs, as well as the good bonding interfaces between the CNTs and the Ag matrix. Additionally, compared with graphite, CNTs have high aspect ratios and a large specific surface area, and are therefore expected to have more of an impact on restraining the dislocation motion and propagation during tensile testing [23]. Finally, the CNT clusters generated a large number of pores, which led to crack propagation during tensile testing. More specific data can be viewed in Table 1.

**Figure 11:**
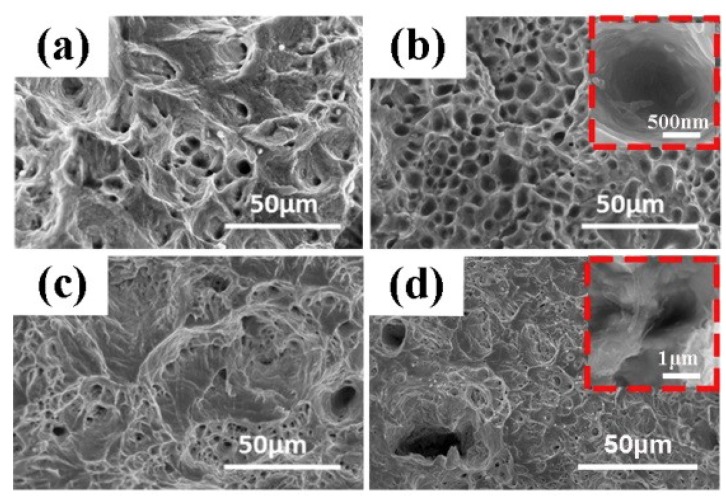
Fracture surface morphologies of the CNTs@Ag/Ag composites with different contents of CNTs by SPS and HP: (**a**) 1 vol.% (SPS), (**b**) 2.5 vol.% (SPS), (**c**) 5 vol.% (SPS), (**d**) 2.5 vol.% (HP).

Figure 11 shows the surface fracture morphologies of the composite materials prepared by SPS after tensile testing. Figure 11b displays the surface morphology of the 2.5 vol.% Ag@CNTs/Ag composite prepared by SPS. Compared with the 1 vol.% Ag@CNTs/Ag composite shown in Figure 11a, Figure 11b exhibits a larger dimple and a more uniform dimple size. The larger dimple indicates that the material absorbs more energy in the process of ductile fracture and has better plastic toughness. When the volume fraction of CNTs reaches 5%, the fracture surface of the composite material has large and obvious cracks, as shown in Figure 11c. It can be seen from Figure 11 that the distribution of CNTs can be determined by the fracture morphology of the composite. CNTs with volume fractions of 1% and 2.5% were uniformly dispersed in the matrix. As the CNTs were dispersed, the material obtained good mechanical properties. When the content of CNTs reached 5 vol.%, there were many CNT aggregates around the dimples, which resulted in many of pores. During the tensile test, the pores are likely to cause crack growth, thus the strength of the composite material decreases significantly. Figure 11d is the fracture morphology of the composite material with a CNT content of 2.5 vol.% prepared by HP, from which it is evident that the CNTs mostly exist in the form of CNT clusters. This will further affect the mechanical properties of the composites. 

### 3.4. Possible Strengthening Mechanisms of CNT@Ag/Ag Composites

The composites prepared by SPS can obtain finer grains under the condition of rapid sintering. In addition, the CNTs in the composite can maintain the characteristics of a complete structure, high strength, and high modulus in the process of rapid sintering. The CNTs can also effectively transfer the applied load from the matrix to the CNTs through the interface in the process of loading, and improve the strength of the composite due to the excellent properties of CNTs. CNTs can also inhibit the growth of grains and produce grain refinement. Therefore, the two main strengthening mechanisms of load transfer and grain refinement are proposed in this study.

#### 3.4.1. Strengthening by Grain Refinement

The strengthening effect of grain refinement can be explained by the Hall-Petch relationship, and the increase of strength can be calculated through the following equation [24]:

(1) $$∆\sigma_{\mathrm{GR}}=K\left(D_{c}^{-0.5}-D_{m}^{-0.5}\right)$$

where $∆\sigma_{\mathrm{GR}}$ is the strength increment brought by grain refinement, K = 0.15 MPa·m^0.5^ for Ag, D_c_ is the average grain sizes of the CNTs@Ag/Ag composite, and D_m_ is the average grain sizes of reference pure Ag. After calculation, the value of $∆\sigma_{\mathrm{GR}}$ is 11.4 MPa.

#### 3.4.2. Strengthening by Load-Transfer Mechanism

The load-transfer mechanism can be estimated using a shear-lag model developed in a previous study [25].

(2) $$\sigma_{L-T}=\sigma_{m}\;\left[V_{f}\frac{S_{\mathrm{eff}}+1}{2}+V_{m}\right]$$

(3) $$S_{\mathrm{eff}}={\mathrm{Scos}}^{2}\theta +\left(1-\frac{4}{3\pi }\right)\left(1+\frac{1}{S}\right)\sin^{2}\theta$$

where $\sigma_{L-T}$ represents the tensile strengthen of the composite, $\sigma_{m}$ represents the tensile strengthening of the Ag matrix (65 MPa), V_f_ is the volume fraction of the CNTs (2.5 vol.%), S_eff_ is the effective aspect ratio of the reinforcements, θ is the misorientation angle between the longitudinal axis of the CNTs and the loading axis of the tensile test, and S is the aspect ratio of the CNTs, the effective aspect ratio of the CNTs is equal to half the aspect ratio of the CNTs when they are randomly distributed (S_eff_ = 0.5S) [9,26]. After calculation, the value of $\sigma_{L-T}$ is 99.8 MPa.

The calculated value of tensile strength of CNTs@Ag/Ag composites was 225.2 MPa, basically consistent with the experimental value of 221 MPa. This indicates that the strengthening mechanisms can be explained based on the effects of the homogeneous dispersion of the CNTs and the load transfer from the matrix to the reinforcements.

## 4. Conclusions

In summary, the dispersion of CNTs is effectively improved by the EPUSA in the reaction process, and the silver nanoparticles are uniformly coated on the CNT surface. The mechanical and electrical properties of the SPS and HP samples are different due to the different levels of CNT agglomeration and grain refinement. Generally, Ag@CNTs/Ag composites produced by the SPS method exhibit better mechanical and electrical properties than their HP counterparts. The strengthening mechanisms mainly refer to the homogeneous dispersion of the CNTs, and the load transfer from the matrix to the reinforcements. 

## Figures and Tables

**Table 1:** Relative density, electrical conductivity and mechanical properties of the composites with different CNTs contents.

Sample		Relative Density (%)	Hardness (HV)	Electrical Conductivity (MS/m)	Ultimate Strength (MPa)	Yield Strength (MPa)	Elongation (%)
SPS (vol.%)	0	98.93	55	62.25	114	65	38.7
	1	98.13	65	61.75	218	112	39.8
	2.5	97.81	73	61.70	221	121	41.6
	5	96.17	92	61.62	116	68	32.1
HP (vol.%)	0	97.96	44	61.57	113	62	41.5
	1	96.74	54	61.29	156	81	37.8
	2.5	96.65	69	61.16	173	89	37.7
	5	95.51	75	60.86	122	72	38.4
